# Predicting the Unpredictable: Prognostic Role of Systemic Inflammatory Indices and Tumor Biology of Neoadjuvant Chemotherapy Response in Gastric and Gastroesophageal Junction Cancer—Insights from a Systematic Review and Real-World Experience

**DOI:** 10.3390/jcm15041484

**Published:** 2026-02-13

**Authors:** Sibel Oyucu Orhan, Bedrettin Orhan, Yağmur Çakır, Seda Sali, Burcu Caner, Birol Ocak, Ahmet Bilgehan Şahin, Adem Deligönül, Erdem Çubukçu, Türkkan Evrensel

**Affiliations:** 1 Department of Medical Oncology, Bursa City Hospital, Bursa 16250, Turkey; seda_ist1987@hotmail.com; 2 Department of Hematology, Bursa Yuksek Ihtisas Training and Research Hospital, University of Health Science, Bursa 16310, Turkey; borhan18@gmail.com; 3 Department of Internal Medicine, Faculty of Medicine, Bursa Uludag University, Bursa 16059, Turkey; yagmurcakir66@gmail.com; 4 Department of Medical Oncology, Aydın Atatürk Public Hospital, Aydın 09020, Turkey; drburcucaner@gmail.com; 5 Department of Medical Oncology, Bursa Yuksek Ihtisas Training and Research Hospital, University of Health Science, Bursa 16310, Turkey; birol08ocak@gmail.com; 6 Department of Medical Oncology, Faculty of Medicine, Bursa Uludag University, Bursa 16059, Turkey; absahin@uludag.edu.tr (A.B.Ş.); ademd@uludag.edu.tr (A.D.); erdemcubukcu@uludag.edu.tr (E.Ç.); evrensel@uludag.edu.tr (T.E.)

**Keywords:** gastric cancer, neoadjuvant chemotherapy, FLOT, prognostic biomarkers, systemic inflammation, lymphovascular invasion

## Abstract

**Background/Objectives:** Perioperative chemotherapy is the standard treatment for locally advanced gastric and gastroesophageal junction adenocarcinoma; however, substantial uncertainty remains regarding the optimal management of non-responding patients and the prognostic relevance of biological and inflammatory biomarkers. This study aimed to determine, using real-world data integrated with a comprehensive literature review, whether long-term survival is driven primarily by the choice of chemotherapy regimen or by the tumor’s intrinsic biological aggressiveness and the host’s systemic inflammatory response. **Methods:** A retrospective analysis was performed of 43 patients with locally advanced gastric cancer who received neoadjuvant chemotherapy. Survival outcomes were stratified by regimen (FLOT versus non-FLOT) and analyzed using Kaplan–Meier methods. The prognostic value of clinicopathological features and systemic inflammatory indices was assessed using multivariate Cox regression models to identify independent predictors of mortality. **Results:** Although FLOT showed a trend toward improved overall survival (OS) (median not reached vs. 18.9 months), this difference did not reach statistical significance. Univariate analysis linked lymphovascular invasion (LVI) (HR = 4.17; *p* = 0.003), pan-cytokeratin (panCK) (HR = 2.44; *p* = 0.032), and monocyte-to-lymphocyte ratio (MLR) (HR = 1.73; *p* = 0.027) with survival. To minimize overfitting, two multivariate models were constructed. The first confirmed LVI (HR = 7.32; *p* < 0.001) and panCK (HR = 4.30; *p* = 0.006) as independent prognostic markers. The second identified MLR (HR = 1.65; *p* = 0.033) and panCK (HR = 2.42; *p* = 0.034) as independent adverse factors. **Conclusions:** Our findings suggest a paradigm shift in prognostic assessment for locally advanced gastric cancer: therapeutic success appears to depend more on underlying tumor biology and the immune microenvironment than on any specific neoadjuvant regimen. High MLR and LVI serve as strong surrogate markers of a biologically aggressive, chemotherapy-resistant phenotype. Consequently, future clinical strategies should move beyond a “one-size-fits-all” chemotherapy approach and prioritize these biomarkers for risk stratification and personalization of multimodal therapy.

## 1. Introduction

Gastric cancer is the fifth most common malignancy worldwide and ranks third among cancer-related causes of death [1]. Because most patients present with locally advanced disease at diagnosis, surgery alone has proven insufficient as a treatment option, leading to the adoption of multimodal approaches [2,3]. International guidelines, such as those from the European Society for Medical Oncology and the National Comprehensive Cancer Network, recommend neoadjuvant or perioperative chemotherapy as the standard treatment for locally advanced gastric and gastroesophageal junction (GEJ) cancers [4].

The first major shift in treatment paradigms in this field came from clinical trials demonstrating a survival benefit with perioperative chemotherapy. The MAGIC trial, conducted by Cunningham and colleagues, established the neoadjuvant approach as standard by showing that perioperative ECF (epirubicin, cisplatin, and 5-fluorouracil) chemotherapy significantly increased OS compared with surgery alone (5-year survival: 36% vs. 23%) [2]. Around the same time, the FNCLCC/FFCD trial from France corroborated the effectiveness of fluorouracil- and cisplatin-based perioperative chemotherapy, demonstrating higher rates of curative (R0) resection (84% vs. 73%) and improved 5-year survival (38% vs. 24%) compared with surgery alone [5].

More recently, the FLOT4-AIO trial advanced the standard of care by showing that the docetaxel-containing FLOT regimen (5-fluorouracil, leucovorin, oxaliplatin, and docetaxel) increased median OS from 35 to 50 months and significantly improved pathologic complete response (pCR) rates compared with the previously accepted ECF and its non-inferior variant ECX (where 5-fluorouracil is replaced by oral capecitabine) regimens [6]. This superiority is attributed not to a technical modification, but to the pharmacologic composition of the regimen, particularly the addition of docetaxel and oxaliplatin, which enhance cytotoxic synergy and dose intensity compared with earlier epirubicin-based protocols [6]. On the basis of these results, the FLOT regimen has been adopted as the current “gold standard” for resectable gastric and GEJ cancers.

In this context, the FLOT protocol, which offers higher survival and pathological response rates than epirubicin-based regimens, has become the standard neoadjuvant therapy in recent years [7]. However, the literature reports that pCR rates remain below 16% despite FLOT therapy [8]. This underscores the clinical importance of identifying in advance those patients most likely to respond to neoadjuvant treatment, because non-responders are not only exposed to chemotherapy-related toxicity but may also face a potentially harmful delay in surgery [9].

Recent studies have investigated the prognostic value of various biomarkers for predicting treatment response, including the CD4/CD8 ratio, IL-6 level, modified Glasgow Prognostic Score (mGPS), hemoglobin/red cell distribution width ratio (HRR), and systemic inflammation indices [8,9,10,11,12]. For example, Skubleny et al. reported that a high CD4+/CD8+ ratio was associated with a better response to FLOT therapy [10]. Similarly, low IL-6 levels before treatment and at the second cycle have been proposed as predictors of pCR [12]. HRR, a novel parameter reflecting systemic immunoinflammatory burden, has been identified as an independent predictor of both disease free survival (DFS) and OS [13].

Studies focusing on elderly patients have reported significant associations between simple biochemical indices such as mGPS and survival [14]. Parameters such as serum albumin level and lymphocyte count have also been shown to play a role in predicting treatment response [15]. In addition to prognostic markers, factors affecting the deliverability of FLOT treatment are clinically important; for instance, high platelet-to-lymphocyte ratio (PLR) values have been associated with poorer treatment adherence [8].

A total neoadjuvant approach (FLOT × 8), in which all cycles are administered preoperatively instead of the conventional 4 + 4 schedule, has been reported to yield higher treatment completion rates and nearly double the pCR rate [16]. Nevertheless, early recurrence remains a significant problem. In particular, pathological features such as ypN3 stage and extracapsular spread are associated with early relapse, and patients with these findings experience markedly shortened survival [13,16].

This study aimed to present real-world data from our center on patients with locally advanced gastric and GEJ cancers who received neoadjuvant therapy, including their treatment responses and survival outcomes. In addition, through a comprehensive review of recent studies in the literature, the study sought to evaluate the effectiveness of neoadjuvant therapy, the significance of pathological response, and factors affecting prognosis (clinicopathological features, inflammatory indices, and novel biomarkers) with the goal of contributing both to personalized treatment selection and to the broader literature. While the response to neoadjuvant therapy constitutes the immediate clinical endpoint, this study explicitly aimed to determine whether these responses translate into long term survival benefits. Therefore, beyond evaluating pathological regression, a primary objective was to analyze the impact of clinicopathological features and inflammatory indices on OS and DFS in a real-world cohort.

## 2. Materials and Methods

Patients with gastric cancer who received neoadjuvant chemotherapy between January 2008 and December 2021 at the Department of Medical Oncology, Bursa Uludağ University Hospital, were retrospectively reviewed. Patients with histologically confirmed locally advanced gastric or gastroesophageal junction adenocarcinoma who received neoadjuvant chemotherapy between January 2008 and December 2021 were eligible for inclusion. The inclusion criteria were: (1) age ≥18 years; (2) confirmation of adenocarcinoma by endoscopic biopsy; (3) absence of distant metastasis at diagnosis (clinical M0); and (4) administration of at least one cycle of neoadjuvant chemotherapy. Patients with a second primary malignancy, incomplete medical records, or those who underwent upfront surgery without neoadjuvant treatment were excluded. Importantly, to reflect real world outcomes, patients who initiated neoadjuvant therapy but did not proceed to surgery due to disease progression, toxicity, or patient refusal were included in the survival analyses (intention-to-treat approach). Age at diagnosis, sex, smoking status, Eastern Cooperative Oncology Group (ECOG) performance status, body mass index (BMI), primary tumor location, clinical T and N stage before treatment, histologic subtype, presence of lymphovascular and perineural invasion, immunohistochemical staining parameters, surgical status, type of surgery, neoadjuvant chemotherapy regimens administered, toxicities related to neoadjuvant treatment, clinical and pathological responses to neoadjuvant therapy, pretreatment biochemical parameters (hemoglobin, red cell distribution width [RDW], neutrophils, monocytes, lymphocytes, platelets, albumin, lactate dehydrogenase [LDH], carcinoembryonic antigen [CEA], carbohydrate antigen 19-9 [CA 19-9], and Ki-67), systemic inflammatory indices, OS, and DFS were evaluated. Neoadjuvant adverse events were defined and graded according to the National Cancer Institute Common Terminology Criteria for Adverse Events, version 4.0. LVI and other histopathological parameters were assessed by experienced pathologists on routine hematoxylin–eosin-stained sections as part of standard clinical practice.

Indices reflecting systemic inflammatory response and nutritional status were calculated from absolute cell counts and biochemical values obtained from pretreatment blood samples using the following formulas: neutrophil-to-lymphocyte ratio (NLR = neutrophils/lymphocytes), platelet-to-lymphocyte ratio (PLR = platelets/lymphocytes), monocyte-to-lymphocyte ratio (MLR = monocytes/lymphocytes), systemic immune-inflammation index (SII = platelets × neutrophils/lymphocytes), systemic inflammation response index (SIRI = neutrophils × monocytes/lymphocytes), pan-immune-inflammation value (PIV = neutrophils × platelets × monocytes/lymphocytes), prognostic nutritional index (PNI = 10 × serum albumin [g/dL] + 0.005 × total lymphocyte count [/mm^3^]), and HRR (hemoglobin/RDW).

Clinical staging at presentation was performed according to the American Joint Committee on Cancer 7th edition staging system [17]. Endoscopy, computerized tomography (CT), and positron emission tomography-computerized tomography (PET-CT) were used for staging both before and after neoadjuvant treatment. Postoperative pathological response was evaluated using the College of American Pathologists Tumor Regression Grade (TRG) system [18]. According to this grading system, TRG0 (pCR) indicates no viable cancer cells; TRG1 (near-complete response) indicates single cells or small clusters of cancer cells; TRG2 (partial response [PR]) indicates residual cancer with a predominant fibrotic stroma; and TRG3 (poor or no response) indicates minimal or no tumor cell kill with extensive residual cancer.

The primary study endpoints, based on real-world differences in survival among patients receiving different chemotherapy regimens, were defined as follows: OS, measured in months from diagnosis to death or, if the patient was alive, to the date of the last outpatient visit; and DFS, measured in months from diagnosis to recurrence or, if no recurrence occurred, to the date of the last follow-up.

### 2.1. Chemotherapy Regimens Used (Alphabetical Order)

CAPEOX: capecitabine and oxaliplatin
CX: cisplatin and capecitabine
DCF: docetaxel, cisplatin, and 5-fluorouracil
DCX: docetaxel, cisplatin, and capecitabine
ECF: epirubicin, cisplatin, and 5-fluorouracil
ECX: epirubicin, cisplatin, and capecitabine
EOX: epirubicin, oxaliplatin, and capecitabine
FLOT: 5-fluorouracil, leucovorin, oxaliplatin, and docetaxel
FOLFOX: 5-fluorouracil, leucovorin, and oxaliplatin

### 2.2. Statistical Analysis

Statistical analyses were performed using IBM SPSS Statistics for Windows, Version 25.0 (IBM Corp., Armonk, NY, USA). Descriptive statistics were presented as counts and percentages for categorical variables and as mean, standard deviation, minimum, maximum, and median for numerical variables. Comparisons of numerical variables between groups were made using Student’s *t*-test when the assumption of normality was satisfied and the Mann–Whitney U test when it was not. Group proportions were compared using the chi-square test. Survival rates were examined using Kaplan–Meier analysis. For systemic inflammatory indices (NLR, PLR, MLR, SII, SIRI, PIV) and HRR, the study cohort was dichotomized into ‘low’ and ‘high’ groups based on the median value of each parameter. This approach was selected to avoid selection bias and to reflect the internal distribution of the cohort. Risk factors were evaluated using Cox regression analysis. The alpha level for statistical significance was set at *p* < 0.05. During construction of multivariate Cox regression models, possible multicollinearity among independent variables was assessed using the variance inflation factor (VIF). To ensure model reliability, variables found to be highly correlated or with VIF values greater than 5, particularly inflammatory indices that mathematically incorporate one another, were not entered into the multivariate analysis simultaneously. When a three-variable model (LVI, pan-cytokeratin [panCK] positivity, MLR) was attempted, numerical instability was observed due to the small sample size and strong discriminative effect; therefore, to reduce the risk of overfitting, two clinically meaningful, separate two-variable models are reported.

## 3. Results

Forty-three patients were included in the study. The demographic and clinical characteristics of the patients are presented in Table 1. Thirteen patients (30.2%) were female and thirty (69.8%) were male. The median age at diagnosis was 60.3 years (range, 32–86). Thirty-three patients (76.7%) were non-smokers and ten (23.3%) were smokers. Among smokers, the mean pack-years was 7.2 (±16.4). The median BMI was 24.5 kg/m^2^ (range, 17.3–36.3). In fourteen cases (32.6%), the cancer was located in the cardia. Other tumor locations were antrum, 8 (18.6%); corpus, 8 (18.6%); GEJ, 4 (9.3%); other locations, 1 (2.3%); and multiple locations, 8 (18.6%). By histologic subtype, 28 patients (65.1%) had adenocarcinoma, 7 (16.3%) had signet-ring cell carcinoma, 3 (7.0%) had mucinous carcinoma, and 5 (11.6%) had a mixed type. Overall mortality after neoadjuvant treatment did not show a significant association with demographic, clinical, or histopathological parameters.

When post-neoadjuvant cases were compared between the alive and deceased groups with respect to pathological invasion findings, molecular markers, and surgical parameters, significant differences were observed for several variables. LVI was significantly associated with mortality (*p* = 0.003). The mortality rate among patients with LVI was 90.9%, whereas it was 36.0% among those without LVI. Similarly, the presence of perineural invasion had a negative impact on survival (*p* = 0.044); 72.2% of cases with perineural invasion belonged to the deceased group. There was no statistically significant difference between signet-ring cell type or mucinous component and outcome (*p* = 0.187 and *p* = 0.728, respectively). Immunohistochemical markers assessed included HER2 (immunohistochemistry [IHC]) scores, MUC1, MUC2, COX-2, and panCK expression. HER2 positivity (2+ or 3+) and MUC1/MUC2 expression were not associated with survival (*p* > 0.05). However, panCK positivity was significantly associated with mortality (*p* = 0.031); 77.3% of panCK-positive cases were in the deceased group. Regarding surgical characteristics, although the survival rate was higher among patients who underwent surgery (alive, 45.9%), the difference did not reach statistical significance (*p* = 0.066). Surgery was performed in 37 patients (86%), while 6 patients (14%) did not undergo surgical intervention. Among those who underwent surgery, palliative surgery was performed in 1 patient. The patient who underwent palliative surgery was deemed inoperable intraoperatively, and only an ostomy was performed without tumor resection. Total gastrectomy was the most commonly performed procedure (78.4%), and there was no difference in survival according to type of surgery (*p* = 1.000). The proportion of patients who underwent D2 lymphadenectomy was 91.9%; mortality in this group was markedly lower, but the difference was not statistically significant (*p* = 1.000). Resection type and surgical margin status were associated with significant differences in survival. Among patients who underwent R0 resection, 57.7% were in the alive group, whereas 80.0% of R1 resections and all R2 resections were in the deceased group (*p* = 0.044). Similarly, the presence of positive surgical margins was strongly associated with mortality (*p* = 0.036). Only 42.3% of patients with negative margins were in the deceased group, whereas this proportion increased to 81.8% among those with positive margins. In lymph node analyses, the mean number of nodes removed was 21.36 ± 12.38. Although the number of nodes removed was higher in the alive group (23.94 ± 12.13 vs. 19.05 ± 12.47), the difference was not statistically significant (*p* = 0.127). By contrast, the number of metastatic lymph nodes was a significant parameter for survival (*p* = 0.019): the median number of metastatic lymph nodes was 0 (range, 0–9) in the alive group and 2 (range, 0–38) in the deceased group. In gastric cancer patients after neoadjuvant therapy, the presence of lymphovascular and perineural invasion, panCK positivity, positive surgical margins, and an increased number of metastatic lymph nodes were significantly associated with mortality (Table 2).

Clinical performance status, treatment regimen, toxicity profile, and response rates were compared between the alive and deceased groups (Table 3). Although ECOG performance status was generally better in the alive group (46.4% with ECOG 0), the difference was not statistically significant (*p* = 0.367). Twenty patients received the FLOT regimen, whereas 23 patients received other chemotherapy protocols. The neoadjuvant chemotherapy regimen was significantly associated with survival (*p* = 0.014). Among patients treated with FLOT, 60.0% were alive compared with 21.7% in the group receiving other regimens.

During neoadjuvant treatment, treatment-related toxicity of any grade was observed in 88.4% of patients (*n* = 38). Most toxicities were manageable; grade 1–2 toxicity occurred in 90.7% (*n* = 39), whereas grade 3–4 toxicity occurred in 18.6% (*n* = 8). No treatment-related mortality (grade 5 toxicity) occurred during the study. Among hematologic adverse events, anemia was the most common toxicity and was observed in 79.1% of patients (*n* = 34); all cases were grade 1–2. Neutropenia developed in 48.8% of patients (*n* = 21), with grade 3 neutropenia in 9.3% (*n* = 4) and grade 4 neutropenia in 7.0% (*n* = 3), yielding a combined grade 3–4 incidence of 16.3% (*n* = 7). Non-hematologic adverse events were less frequent. Neuropathy and diarrhea each occurred in 4.7% of patients (*n* = 2), and all cases were low grade (grade 1–2). Treatment discontinuation due to toxicity was documented in 4.7% of patients (*n* = 2) (Table 3).

The presence of neoadjuvant toxicity was less frequent in the alive group (alive: 34.2% vs. deceased: 65.8%), and this trend approached statistical significance (*p* = 0.071). When grade 1–2 toxicity rates were examined, all patients without any toxicity were in the alive group (*p* = 0.019). However, there was no significant association between grade 3–4 toxicity and survival (*p* = 1.000). Anemia emerged as another important parameter associated with survival (*p* = 0.016). As the severity of anemia increased, the proportion of deceased patients increased markedly; 81.8% of patients with grade 2 anemia were in the deceased group. In contrast, no significant differences in survival were observed for neutropenia, neuropathy, or diarrhea (all *p* > 0.05). Regarding clinical response to neoadjuvant therapy, the alive group had a PR rate of 50.0%, whereas all patients with progressive disease and stable disease in the deceased group experienced fatal outcomes (*p* = 0.010). There was one patient with complete response (CR), who was alive at the time of analysis. Similarly, pathological response was more frequent in the alive group (CR + PR: 91.9%). Cases with poor or no pathological response had a mortality rate of 90.9%, and this difference was statistically significant (*p* = 0.042). In this cohort, the FLOT chemotherapy regimen, combined with a favorable toxicity profile and good clinical and pathological responses, was associated with improved survival. By contrast, patients unresponsive to treatment and exhibiting high toxicity accompanied by grade 2 anemia and LVI were strongly associated with mortality (Table 3).

When laboratory parameters and inflammatory markers at diagnosis were evaluated, hemoglobin levels were similar between the alive and deceased groups (alive: 12.05 ± 2.49 g/dL; deceased: 12.03 ± 1.84 g/dL; *p* = 0.973). There were no significant differences between groups in RDW or in neutrophil, monocyte, lymphocyte, or platelet counts (all *p* > 0.05). The mean neutrophil count was 5517 ± 2228/mm^3^ and the mean lymphocyte count was 2475 ± 3206/mm^3^. Mean serum albumin was 3.95 ± 0.47 g/dL and did not differ between groups (*p* = 0.636). Although LDH levels tended to be higher in the deceased group, this difference did not reach statistical significance (246.6 ± 275.0 vs. 185.8 ± 33.8; *p* = 0.813). In contrast, CEA and CA 19-9 levels were significantly associated with survival. Mean CEA was 2.43 ± 2.28 ng/mL in the alive group and 14.58 ± 33.92 ng/mL in the deceased group (*p* = 0.014). Similarly, mean CA 19-9 was 82.0 ± 175.4 U/mL in the alive group and 292.9 ± 449.7 U/mL in the deceased group (*p* = 0.044). These findings suggest that elevated tumor markers are associated with poorer prognosis. The Ki-67 proliferation index did not differ between groups (alive: 63.5 ± 18.7; deceased: 55.8 ± 24.7; *p* = 0.574). On evaluation of post-neoadjuvant CEA and CA 19-9 values, a significant difference was detected for CA 19-9 (*p* = 0.040), with higher levels observed in the deceased group. No significant differences were observed between groups for inflammatory indices (NLR, PLR, MLR, SII, SIRI, PNI, PIV, HRR) (all *p* > 0.05). Overall, high pre-therapeutic CEA and CA 19-9, as well as elevated post-neoadjuvant CA 19-9, were significantly associated with increased mortality, whereas inflammatory indices appeared to be complementary but not independent predictors of survival. In this cohort, CEA and CA 19-9 levels were the main determinants of survival, while serum albumin, LDH, and the inflammatory indices (NLR, PLR, SII, PNI, HRR) did not show significant differences between groups (Table 4).

**Figure 1:**
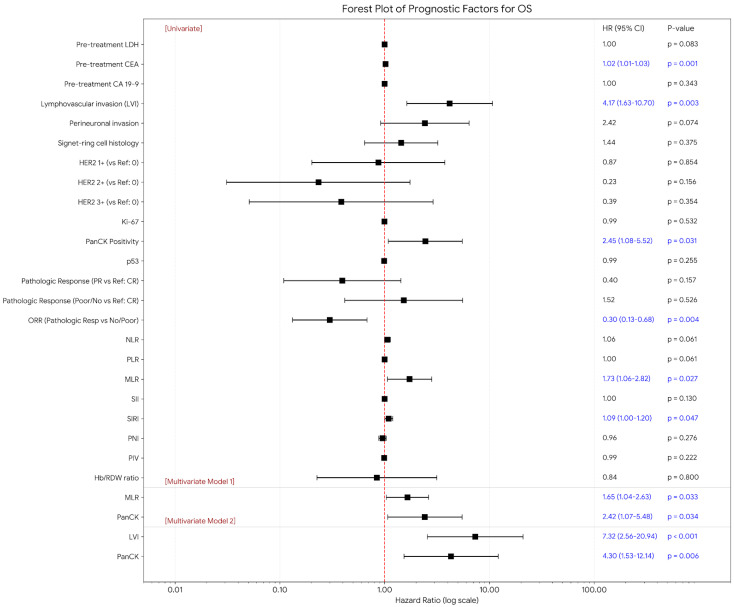
Forest plot of univariate and multivariate Cox regression analyses for overall survival. Only statistically significant confidence intervals are displayed for clarity. Model 1 includes MLR and panCK, whereas Model 2 includes LVI and panCK. HR, Hazard ratio; CI, Confidence interval; CT, Chemotherapy; CR, Complete response; PR, Partial response; PD, Progressive disease; NLR, Neutrophil-to-lymphocyte ratio; PLR, Platelet-to-lymphocyte ratio; MLR, Monocyte-to-lymphocyte ratio; ORR, Overall Response Rate; SII, Systemic immune-inflammation index; SIRI, Systemic inflammation response index; PNI, Prognostic nutritional index; PIV, Pan-immune-inflammation value.

With respect to demographic and baseline clinical variables age at diagnosis, sex, smoking status, and BMI were not significantly associated with OS. In univariate analysis, pretreatment CEA level was associated with OS (HR = 1.02; *p* = 0.001), while LDH demonstrated a non-significant trend toward poorer survival. Among pathological features, LVI emerged as a strong adverse factor (HR = 4.17; *p* = 0.003). Similarly, panCK positivity was independently associated with increased mortality risk (HR = 2.45; *p* = 0.031). In terms of pathological response, poor or absent response was significantly associated with worse outcomes (HR = 0.30; *p* = 0.004). Among systemic inflammatory indices, MLR (HR = 1.73; *p* = 0.027) and SIRI (HR = 1.09; *p* = 0.047) were significant predictors in univariate analysis, whereas other indices did not reach statistical significance. To account for sample size limitations and reduce overfitting, two separate multivariable models were constructed. In Model 1, both MLR and panCK remained independent adverse prognostic factors. In Model 2, LVI emerged as the strongest independent determinant of mortality, increasing risk approximately seven-fold, while panCK positivity also retained independent prognostic significance (Figure 1).

**Figure 2:**
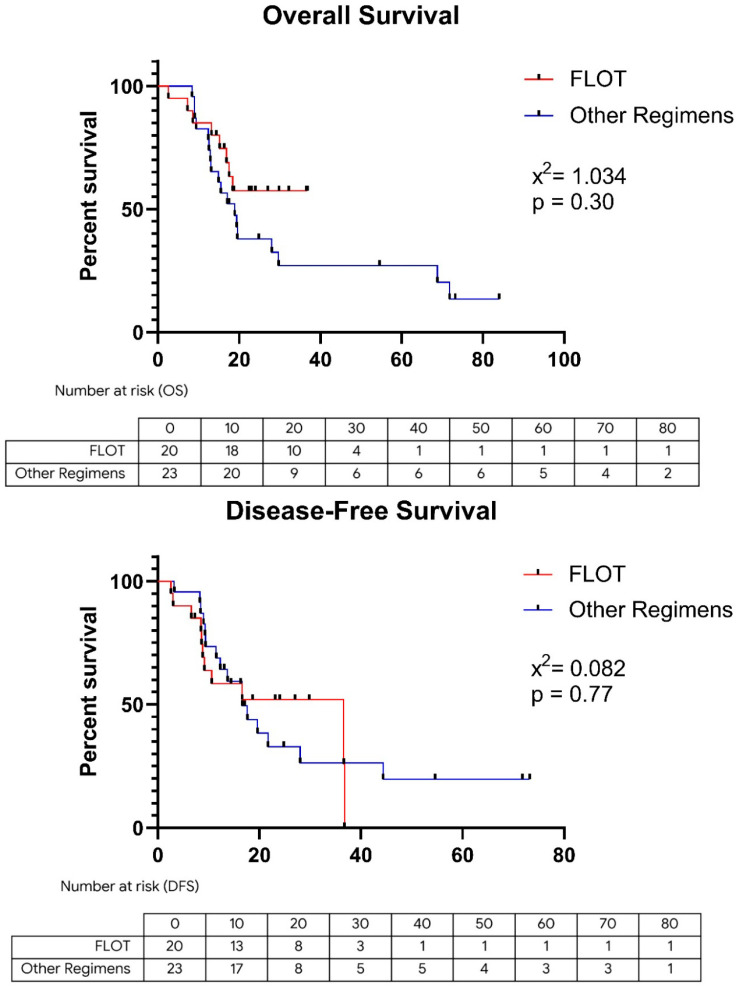
Comparison of Overall and Disease Free Survival between FLOT and Other Regimens. Kaplan–Meier curves demonstrate no statistically significant difference in Overall Survival (*p* = 0.30) or Disease-Free Survival (*p* = 0.77) between the FLOT (red line) and Other Regimens (blue line) groups. *p*-values were calculated using the Log-rank test.

Kaplan–Meier analyses illustrating the effect of neoadjuvant chemotherapy regimens on survival are presented in Figure 2. For OS, the median was not reached in the FLOT group, whereas the median OS in the non-FLOT group was 18.9 months. Although a clinically apparent survival advantage was observed in the FLOT arm, this difference did not reach statistical significance (*p* = 0.30). Similarly, DFS numerically favored the FLOT regimen: median DFS was 36.6 months in the FLOT group versus 16.6 months in the non-FLOT group, but this difference was not statistically significant (*p* = 0.77).

## 4. Discussion

The perioperative application of the FLOT regimen in locally advanced gastric and GEJ cancers has been accepted as the standard treatment approach since the landmark results of the FLOT4-AIO trial [15]. This regimen has demonstrated higher pathological response rates and superior survival outcomes compared with older regimens such as ECF/ECX [15]. In the present study, we sought to evaluate clinicopathological and inflammatory markers associated with pathological response and survival in a cohort of 43 patients who received neoadjuvant therapy. The most notable finding of our study is that the principal determinants of post-neoadjuvant survival appear to be pathological and hematological parameters that reflect tumor biological aggressiveness and the host inflammatory state, rather than the specific chemotherapy regimen administered. In univariate analyses, LVI (HR ≈ 4.2), panCK positivity (HR ≈ 2.4), and elevated MLR (HR ≈ 1.7) were significantly associated with mortality.

Given the limited sample size and the need to restrict the number of covariates, we elected to construct two separate two-variable multivariate models instead of a single three-variable model. In the model that included LVI and panCK, LVI emerged as the strongest independent predictor, increasing mortality risk approximately seven-fold, while panCK positivity remained an independent adverse prognostic factor. Similar findings have been reported in prior cohorts, in which LVI was associated with an increased risk of relapse, likely reflecting a higher likelihood of occult tumor cell dissemination [19,20]. In the second model, which included MLR and panCK, both variables retained independent prognostic significance. Beyond presenting real-world data from our center, this study also integrates findings from 19 recent large-scale studies published after the FLOT trials [6,7], enabling a broader evaluation of whether treatment success is primarily determined by intrinsic tumor biology and systemic inflammatory profile rather than regimen selection. A comparative overview of these contemporary studies is provided in Table 5.

The randomized phase II DRAGON III trial, conducted in Asian populations, showed no significant difference between the SOX (S-1 plus oxaliplatin) regimen and FLOT in long-term survival (OS and DFS) (*p* > 0.75) [21]. Similarly, a propensity-score–matched analysis by Zhang et al. reported no survival difference between FLOT and the DOS (docetaxel, oxaliplatin, S-1) regimen [22]. The RESOLVE trial likewise demonstrated the efficacy of the perioperative SOX regimen and reported comparable survival outcomes between SOX and CAPEOX [23]. The Europe-based CRITICS trial emphasized the low completion rate of postoperative treatment (approximately 50%) and reinforced the concept that the majority of systemic therapy should ideally be delivered preoperatively [24]. A large Turkish series by Başoğlu et al. also reported no significant survival differences among regimens such as FLOT, DCF, and ECF, supporting the notion that biology may be more important than regimen choice [25]. Although our study did not identify a statistically significant survival difference between patients receiving FLOT and those receiving non-FLOT regimens (*p* > 0.05), the Kaplan–Meier curves (Figure 2) demonstrate a clear trend toward improved outcomes with FLOT. Notably, the median OS had not been reached in the FLOT group, whereas it was 18.9 months in the comparator group, supporting the potential efficacy of FLOT despite the limited sample size. Heterogeneous treatment responses and the fact that a substantial proportion of patients are unable to complete adjuvant therapy after surgery underscore the urgent need for prognostic and predictive biomarkers that can identify treatment failure early and optimize patient selection [14,16,26].

Although response to neoadjuvant therapy is widely regarded as one of the most important predictors of survival, consensus in the literature remains incomplete. Sah et al. reported that survival was determined primarily by the degree of pathologic response (TRG) rather than by the chemotherapy regimen used (*p* = 0.020) [21]. In the present study, patients who achieved a favorable pathological response (*p* = 0.042) and, in particular, those treated with FLOT (*p* = 0.014) demonstrated a survival advantage, even though this did not reach statistical significance across all comparisons. Our findings illustrate a numerical survival trend rather than definitive statistical superiority. However, a recent real-world analysis by Eroğlu et al., comparing Turkish and German cohorts (*n* = 102), found that major pathologic response (mPR) was not independently associated with either OS or DFS, indicating that intrinsic tumor aggressiveness may be more determinant than pathological response alone [27]. In contrast, Başoğlu et al., in a large case series, reported that non-responders had a 2.16-fold higher risk of death compared with responders (HR = 2.16; *p* < 0.001), reinforcing the independent prognostic value of pathological response [25]. Furthermore, achieving pCR has been associated with nearly 100% 3-year survival rates in several studies [26,28].

Understanding the fundamental causes of treatment failure is critical. Hui et al. reported that among patients who did not achieve pCR, the predominant problem was not local recurrence (8.4%) but distant metastasis, which occurred in 41.0% of cases [28]. In the present study, the strong association of LVI, perineural invasion, and metastatic lymph node count (*p* = 0.019) with survival supports this tendency toward systemic dissemination. Consistently, Başoğlu et al. showed that the most common recurrence sites were the peritoneum (11.1%) and liver (6.2%), confirming that systemic failure predominates over local recurrence (2.9%) [25]. When predictors of early relapse were evaluated, Rompen et al. identified advanced pathological stage (ypT3–4) and nodal positivity (ypN+) as the strongest determinants of “early recurrence” within 18 months after surgery [14]. Eroğlu et al. further demonstrated that pathological lymph node positivity (ypN+) is the most powerful independent prognostic factor for survival (HR: 4.45–6.38), irrespective of ethnicity [27]. In addition, Dayanamby et al. reported that extracapsular nodal spread (*p* = 0.009) is an independent marker of early recurrence [13]. The substantial impact of LVI on mortality (HR ≈ 4.17) suggests that it is not simply a marker of local extension but rather histopathologic evidence of occult micrometastatic disease. LVI reflects the ability of tumor cells to enter the vascular/lymphatic circulation, seed distant niches that may be relatively resistant to chemotherapy, and ultimately drive systemic progression [19]. Current treatment guidelines place increasing importance on programmed death-ligand 1 (PD-L1) expression and microsatellite instability/deficienct mismatch repair (MSI/dMMR) status when estimating the potential benefit from immunotherapy in gastric cancer [29]. Our retrospective cohort, however, represents a time period in which these biomarkers were not yet routinely incorporated into clinical practice. Their absence in our dataset therefore underscores the practical value of readily available systemic inflammatory indices such as MLR. Prospective studies integrating conventional histopathological parameters with emerging molecular subtypes may provide a more comprehensive prognostic framework in the future.

In light of these challenges, the concept of total neoadjuvant therapy (TNT) has gained interest as an alternative to the standard perioperative 4 + 4 FLOT approach. Rencuzogullari et al. reported that delivering all eight FLOT cycles preoperatively (FLOT × 8) significantly increased treatment completion rates from 67.6% to 89.1% (*p* < 0.01) without increasing surgical morbidity [16]. Yang et al. also observed that TNT tended to increase pCR rates (14% vs. 5.8%), although it did not translate into a statistically significant benefit in OS or RFS [26]. However, treatment intensification warrants caution. Eroğlu et al. reported that among patients who received more than four preoperative FLOT cycles, survival did not improve and instead worsened (HR: 3.20, *p* = 0.021) [27]. This finding may be related to prolonged preoperative delays to surgery or the selection of chemotherapy-resistant tumor clones.

The effectiveness of FLOT may also vary according to histologic subtype. Giampieri et al. demonstrated that patients with signet-ring cell (SRC) histology had significantly worse DFS and OS when treated with perioperative FLOT compared with those undergoing surgery followed by adjuvant therapy [30]. Similarly, Eroğlu et al. confirmed SRC histology as an independent risk factor for poor OS (HR: 2.59) [27]. These findings suggest that SRC histology may be relatively resistant to neoadjuvant chemotherapy and that a surgery-first strategy should be considered in this subgroup.

The heterogeneity of treatment response has prompted investigation into biomarkers that reflect systemic inflammation and nutritional status. Monti et al. demonstrated that a high pre-treatment PLR was the strongest risk factor for treatment discontinuation (non-adherence) (OR: 5.03), whereas sarcopenia did not affect adherence [8]. In the present study, inflammatory indices such as elevated MLR and SIRI were associated with poor prognosis, supporting the prognostic value of markers reported in the literature, including mGPS, HRR, PLR, and NLR [11,12,14,31,32]. Yilmaz et al. reported that high postoperative Ki-67 was an independent risk factor for OS [31]. By contrast, Ürün et al. suggested that high NLR and low lymphocyte count might be associated with better treatment response, a finding that contradicts the broader consensus in the literature [32]. From a hematologic perspective, it is not surprising that the MLR emerged as an independent prognostic factor in our cohort. Elevated peripheral monocyte counts likely reflect the density of tumor-associated macrophages within the tumor microenvironment. In particular, M2-polarized macrophages promote angiogenesis and suppress antitumor immunity by secreting vascular endothelial growth factor, epithelial growth factor and matrix metalloproteinases [33]. Conversely, lymphopenia indicates an inadequate cellular immune response against the tumor. Therefore, a high MLR is not merely a numeric ratio but rather a systemic reflection of active “immune escape” mechanisms and the presence of a pro-tumor inflammatory environment. Beyond these standard indices, next-generation biomarkers also show promise. Marcisz-Grzanka et al. reported that low IL-6 levels measured before the second treatment cycle were strongly associated with pCR [10]. In the context of the immune microenvironment, Skubleny et al. demonstrated that a high pre-treatment CD4/CD8 T-cell ratio predicted good response [9]. Mirshahvalad et al. further reported that FDG-PET/CT parameters (SULpeak and SUVmax) may be useful for predicting both pathologic response and survival [34].

**Table 5:** Comparison of studies evaluating pathologic response, OS, and DFS in patients with gastroesophageal and/or gastric cancer receiving neoadjuvant therapy.

Study (Author, Year)	Number of Patients (N)	Treatment Regimen	Median OS/DFS (or Follow-Up)	Pathologic Complete Response (pCR)	Prognostic Factors (*p* < 0.05)
Yılmaz A et al. (2019) [12]	85	Neoadjuvant FLOT	Median Follow-up: 30 months; Mean OS: 45.9 months; Mean DFS: 32 months	11.8%	Low NLR, low SII, and high HRR associated with longer DFS/OS; only HRR remained independent.
Yılmaz H et al. (2021) [31]	75	Neoadjuvant FLOT	Median follow-up: 26.5 months; Mean OS: 34.1 months; Mean DFS: 30 months	34.7%	Post-NAC Ki-67 and pre-NAC PLR associated with poor OS; post-NAC PLR associated with poor DFS.
Basoglu et al. (2022) [25]	794	FLOT (65%), DCF, ECF, FOLFOX, CF/CX	Estimated median OS: 58.4 months; Estimated median DFS: 50.7 months; Median follow up: 16 months (1–154 months) Estimated 3 year OS: 59%	8.2% (FLOT) vs. 5.1% (others) (*p* = 0.14, not significant) R0 resection: 84%	Pathologic non-response and positive margins (R1/R2) (*p* < 0.001).
Giampieri R et al. (2023) [30]	76 (32% SRC)	Perioperative FLOT vs. surgery + adjuvant CT	Median follow-up: 39 months; Median OS: 37.3 months; Median DFS: 26.3 months	10% (6/66)	SRC histology, cT4, and R2 resection predicted worse OS/DFS.
Yang J et al. (2023) [26]	149 (121 periop; 28 TNT)	Perioperative vs. TNT (FLOT 31% vs. 79%)	Median follow-up: 50 months (periop), 31 months (TNT), no significant difference in OS/RFS (*p* > 0.1)	14% (TNT) vs. 5.8% (periop) (*p* = 0.6, not significant)	All 11 patients who achieved pCR had no recurrence, and 3-year OS was 100%.
Paszt A et al. (2023) [15]	88 (52 FLOT vs. 36 ECF/ECX)	Neoadjuvant FLOT vs. ECF/ECX	Median follow-up: 26 months	pCR (TRG 1): significantly higher in FLOT (13.95% vs. 9.09%, *p* = 0.042)	FLOT regimen achieved better pCR. Mean number of positive LNs was significantly lower in the FLOT group (1.35 vs. 5; *p* = 0.0267).
Mirshahvalad SA et al. A et al. (2023) [34]	31 (21 gastric, 10 GE)	Perioperative FLOT	Median follow-up: 42 months; Median PFS: 60 months	Response (Becker Ia, Ib, II): 65%	Pre-NAC SULpeak (PET) predicted pathologic response (*p* = 0.03); 80% probability at a cut-off of 4.
Skubleny D et al. (2023) [9]	18 (neoadjuvan)	Neoadjuvant FLOT	(No data)	Response (pCR/near-pCR): 22% (4/18)	A high pre-treatment CD4/CD8 ratio predicted good treatment response (*p* = 0.025).
Melekoglu E et al. (2023) [11]	71 (geriatric, >65 years)	Perioperative FLOT	Median follow-up: 60 months (mGPS 0), 15 months (Group 1), 38 months (Group 2); 3-year OS: 84.4% (mGPS 0) vs. 30.6% (mGPS 2)	33.8% (24/71)	Higher mGPS (1–2) was associated with significantly worse OS (*p* < 0.001). High BMI was a risk factor for mGPS 2 (*p* = 0.03).
Monti M et al. (2023) [8]	84	Mixed regimens (FLOT, DOC, FOLFOX, CF, ECX, ECF, EOX)	Median follow-up: 44.8 months; treatment adherence was not associated with OS/PFS (*p* > 0.1)	Treatment adherence was not associated with pathologic response (*p* = 0.281)	High PLR was a strong risk factor for treatment discontinuation (OR: 5.03, *p* = 0.017).
Dayanamby A et al. (2024) [13]	196	Neoadjuvant FLOT	1-year OS: 90.8%; 1-year RFS: 83.7%; early recurrence (<12 months): 13.8%	pCR (TRG 1): 12% (no early recurrence in pCR cases)	Risk factors for early recurrence: extracapsular spread and ypN3 stage.
Hui C et al. (2024) [28]	60	Preoperative CT (FLOT 38.6%, FOLFOX 30%, others; ECF/ECX/EOX 23.3%)	3-year OS: 62.3% overall; CR 100%, PR 64%, NR 55.6%; 3-year PFS: 53% overall; CR 100%, PR 53%, NR 44.4%	pCR: 6.7% (*n* = 4)	Those achieving pCR had 100% 3-year OS/PFS. Main failure pattern among non-pCR patients: distant metastasis (41.0%) vs. locoregional recurrence (8.4%).
Marcisz-Grzanka K et al. (2024) [10]	61	Neoadjuvant FLOT	No data	pCR (TRG 1a/b + ypN0): 21%	Low IL-6 (before cycle 2) was significantly associated with pCR (*p* = 0.004).
Rompen IF et al. (2025) [14]	334	Neoadjuvant FLOT (84%) or CROSS (16%)	Median follow-up: 38.4 months; early recurrence (<18 months): mSAR 9.1 months (vs 17.8 months for late recurrence, *p* = 0.039)	Major response rate: 35%	Risk factors for early recurrence: ypT3–4 (*p* = 0.006) and ypN+ (*p* = 0.013).
Rencuzogullari A et al. (2025) [16]	74 (matched)	Perioperative FLOT (4 + 4) vs. total neoadjuvant (TNT) FLOT (×8)	Median follow-up: No significant difference in 36-month OS/DFS (*p* > 0.05)	pCR: 18.9% (FLOT × 8) vs. 8.1% (FLOT 4 + 4) (*p* = 0.25, not significant) All pCR patients (*n* = 10) remained disease-free and alive (*p* < 0.001).	Treatment completion was significantly higher with FLOT × 8 (89.1% vs. 67.6%, *p* < 0.01).
Ürün YY et al. (From Ucgul, 2025) [32]	91 (Geriatric)	Neoadjuvant FLOT	Median follow-up: 28.7 months (12.3–48.1). Predicted DFS: 43.4 vs. 18.7 months (responsive vs. non-responsive); predicted OS: 37.4 vs. 19.9 months	24.1% (22/91) (Becker TRG 1 + 2)	High albumin, low lymphocytes, and high NLR were associated with positive treatment response.
Sah et al. (2025) [21]	74 (40 FLOT vs. 34 SOX)	Neoadjuvant FLOT vs. SOX	Median follow-up: 65.7 months; mOS (*p* = 0.76), mDFS (*p* = 0.84)	TRG 1a/1b: 20% (FLOT) vs. 32.4% (SOX)	Pathologic response (TRG) was strongly associated with survival (*p* = 0.020). Gastrectomy type was an independent prognostic factor (*p* = 0.002); regimen choice was not significant.
Eroglu, et al. (2025) [27]	102 (47 Turkish, 55 German)	Perioperative FLOT	Median OS: 57.9 vs. 29.3 months (German vs. Turkish, *p* = 0.005); median DFS: 53.2 vs. 21.4 months (*p* = 0.03)	mPR (major pathologic response; TRG 1a + 1b): 36.0% (German) vs. 18.5% (Turkish) (*p* = 0.04). mPR was not associated with OS or DFS.	Pathologic lymph node positivity (HR: 6.45; ypN+): the strongest independent risk factor for OS (*p* = 0.022) and DFS. Intensified treatment (>4 cycles of neoadjuvant FLOT): independently associated with poor OS (HR: 3.34; *p* = 0.032).
Current study	43	Neoadjuvant therapy (including FLOT)	Median OS: 19.4 months; median follow-up: 29.8 months	pCR/good response (*p* = 0.042) was associated with survival	LVI, FLOT regimen, MLR, SIRI, panCK.

Abbreviations: Albumin, Serum albumin; BMI, Body mass index; CF, Cisplatin plus 5-fluorouracil; CROSS, Chemoradiotherapy for Oesophageal Cancer Followed by Surgery Study regimen; DOC, Docetaxel-based regimen; ECF, Epirubicin, cisplatin, and 5-fluorouracil; ECX, Epirubicin, cisplatin, and capecitabine; EOX, Epirubicin, oxaliplatin, and capecitabine; ER, Early recurrence; FLOT, 5-Fluorouracil, leucovorin, oxaliplatin, and docetaxel; FOLFOX, 5-Fluorouracil, leucovorin, and oxaliplatin; GE, Gastroesophageal; HR, Hazard ratio; HRR, Hemoglobin-to-red cell distribution width ratio; IL-6, Interleukin-6; LN, Lymph node; LVI, Lymphovascular invasion; mGPS, Modified Glasgow Prognostic Score; mPR, Major pathologic response; mSAR, Median survival after recurrence; NAC, Neoadjuvant chemotherapy; NLR, Neutrophil-to-lymphocyte ratio; NR, No response; OS, Overall survival; panCK, Pan-cytokeratin; PFS, Progression-free survival; PET, Positron emission tomography; PLR, Platelet-to-lymphocyte ratio; PR, Partial response; pCR, Pathologic complete response; pN, Pathologic nodal stage; R0, Complete resection (negative margins); R1, Microscopic residual tumor; R2, Macroscopic residual tumor; RFS, Recurrence-free survival; SII, Systemic immune-inflammation index; SIRI, Systemic inflammation response index; SOX, S-1 plus oxaliplatin; SRC, Signet-ring cell carcinoma; SULpeak, Peak standardized uptake value; TNT, Total neoadjuvant therapy; TRG, Tumor regression grade; ypN, Post-treatment pathologic nodal stage; ypT, Post-treatment pathologic T stage.

### Limitations

This study has several limitations, including its retrospective design and relatively small sample size, which may have reduced statistical power, particularly for subgroup analyses. The data collection period also encompassed years preceding the widespread adoption of the FLOT regimen, resulting in a cohort treated with heterogeneous chemotherapy protocols. The retrospective nature of our data meant that modern molecular markers, such as PD-L1 and MSI status, were not available for the entire cohort. This limits the ability to correlate our findings with the latest molecular classification systems. Furthermore, the absence of contemporary targeted therapies and immunotherapeutic agents, both of which now play an increasing role in gastric cancer management, limits the comparability of our findings with current treatment algorithms.

## 5. Conclusions

In conclusion, although the survival difference between FLOT and other regimens did not reach statistical significance (*p* = 0.30) in our cohort, the fact that median OS was not reached in the FLOT arm (compared with 18.9 months in the comparator arm) indicates a clear trend toward improved survival with FLOT. Taken together with the relevant literature, these findings suggest that long-term outcomes are driven less by the specific chemotherapy regimen and more by the tumor’s intrinsic biological aggressiveness and pathologic response. In particular, LVI appears to be the strongest independent determinant of poor prognosis. While the sample size is limited, the high significance of LVI (*p* < 0.001) in this cohort suggests a robust biological effect that transcends sample size constraints. Although not diagnostic on their own, systemic inflammatory indices such as MLR, whose prognostic value was confirmed in the present study, may serve as useful risk-stratification tools when interpreted alongside tumor-related risk factors, helping to identify patients who are likely to have suboptimal responses to standard therapy. While comprehensive molecular profiling, including MSI and PD-L1 status, is likely to shape the future of personalized therapy, our results indicate that readily available parameters such as LVI and MLR continue to provide meaningful prognostic information in routine clinical practice, particularly in resource-limited settings. Accordingly, future clinical strategies should focus on the early identification of high-risk patients through the combined use of biological, inflammatory, and molecular markers, with an emphasis on individualized treatment algorithms rather than uniform treatment intensification. However, given the limited sample size and the historical nature of part of the cohort, these findings should be interpreted with appropriate caution. The study was conducted at a single tertiary referral center; therefore, external validation using multicenter datasets is required for broader generalizability.

## Figures and Tables

**Table 1:** Relationship between clinicopathological variables and outcomes in neoadjuvant gastric cancer cases.

		Total *n* = 43	Alive *n* = 17 (39.5%)	Deceased *n* = 26 (60.5%)	*p*
**Sex, *n* (%)**	Female	13 (30.2%)	7 (53.8%)	6 (46.2%)	0.310
	Male	30 (69.8%)	10 (33.3%)	20 (66.7%)	
**Age at diagnosis** Mean ± SD/Median (Min–Max)		60.47 ± 10.58 60.35 (32–86)	61.35 ± 6.66 60.35 (50–70)	59.89 ± 12.60 60.64 (32–86)	0.571
**Smoking status, *n* (%)**	Non-smoker	33 (76.7%)	14 (42.4%)	19 (57.6%)	0.714
	Smoker	10 (23.3%)	3 (30.0%)	7 (70.0%)	
**Smoking (pack-years)** Mean ± SD/Median (Min–Max)		7.28 ± 16.40 0 (0–80)	4.71 ± 11.25 0 (0–40)	8.96 ± 19.06 0 (0–80)	0.460
**Baseline weight (kg)** Mean ± SD/Median (Min–Max)		70.74 ± 14.27 69 (39–105)	70.82 ± 12.07 73 (47–84)	70.69 ± 15.78 67.5 (39–105)	0.637
**Baseline BMI** Mean ± SD/Median (Min–Max)		25.49 ± 4.73 24.5 (17.3–36.3)	26.00 ± 4.10 27.4 (17.8–32.5)	25.15 ± 5.16 23.65 (17.3–36.3)	0.571
**Tumor location, *n* (%)**	Antrum	8 (18.6%)	4 (50.0%)	4 (50.0%)	0.887
	Cardia	14 (32.6%)	4 (28.6%)	10 (71.4%)	
	Corpus	8 (18.6%)	4 (50.0%)	4 (50.0%)	
	GE Junction	4 (9.3%)	2 (50.0%)	2 (50.0%)	
	Other	1 (2.3%)	0 (0.0%)	1 (100.0%)	
	Multiple locations	8 (18.6%)	3 (37.5%)	5 (62.5%)	
**Clinical T stage, *n* (%)**	2a	2 (4.7%)	0 (0.0%)	2 (100.0%)	0.379
	3	27 (62.8%)	13 (48.1%)	14 (51.9%)	
	4a	13 (30.2%)	4 (30.8%)	9 (69.2%)	
	4b	1 (2.3%)	0 (0.0%)	1 (100.0%)	
**Clinical N stage, *n* (%)**	N0	4 (9.3%)	3 (75.0%)	1 (25.0%)	0.284
	N1	39 (90.7%)	14 (35.9%)	25 (64.1%)	
**Staging laparoscopy performed, *n* (%)**	No	39 (90.7%)	15 (38.5%)	24 (61.5%)	1.000
	Yes	4 (9.3%)	2 (50.0%)	2 (50.0%)	
**Histologic subtype, *n* (%)**	Adenocarcinoma	28 (65.1%)	13 (46.4%)	15 (53.6%)	0.260
	Signet-ring cell	7 (16.3%)	1 (14.3%)	6 (85.7%)	
	Mucinous	3 (7.0%)	2 (66.7%)	1 (33.3%)	
	Mixed	5 (11.6%)	1 (20.0%)	4 (80.0%)	
**ypT stage, *n* (%)**	T0	2 (5.6%)	2 (100.0%)	0 (0.0%)	0.057
	T1b	2 (5.6%)	2 (100.0%)	0 (0.0%)	
	T2	2 (5.6%)	2 (100.0%)	0 (0.0%)	
	T3	26 (72.2%)	10 (38.5%)	16 (61.5%)	
	T4	2 (5.6%)	1 (50.0%)	1 (50.0%)	
	T4a	2 (5.6%)	0 (0.0%)	2 (100.0%)	
**ypN stage, *n* (%)**	N0	18 (50.0%)	12 (66.7%)	6 (33.3%)	0.119
	N1	7 (19.4%)	2 (28.6%)	5 (71.4%)	
	N2	3 (8.3%)	1 (33.3%)	2 (66.7%)	
	N3	4 (11.1%)	1 (25.0%)	3 (75.0%)	
	N3a	1 (2.8%)	1 (100.0%)	0 (0.0%)	
	N3b	3 (8.3%)	0 (0.0%)	3 (100.0%)	

Abbreviations: *n*, Number of patients; SD, Standard deviation; Min, Minimum; Max, Maximum; BMI, Body mass index; GE, Gastroesophageal; ypT, Pathologic tumor stage; ypN, Pathologic lymph node stage.

**Table 2:** Comparison of pathological invasion, molecular markers, and surgical parameters with survival in patients receiving neoadjuvant therapy.

		Total	Alive	Deceased	*p*
**Lymphovascular invasion, *n* (%)**	No	25 (69.4%)	16 (64.0%)	9 (36.0%)	**0.003**
	Yes	11 (30.6%)	1 (9.1%)	10 (90.9%)	
**Perineural invasion, *n* (%)**	No	18 (50.0%)	12 (66.7%)	6 (33.3%)	**0.044**
	Yes	18 (50.0%)	5 (27.8%)	13 (72.2%)	
**Signet-ring cell component, *n* (%)**	No	30 (69.8%)	14 (46.7%)	16 (53.3%)	0.187
	Yes	13 (30.2%)	3 (23.1%)	10 (76.9%)	
**Mucinous component, *n* (%)**	No	15 (41.6%)	7 (50.0%)	7 (50.0%)	0.728
	Yes	21 (58.4%)	8 (38.1%)	13 (61.9%)	
**HER2 (IHC), *n* (%)**	0	32 (74.4%)	10 (31.2%)	22 (68.8%)	0.289
	1+	4 (9.3%)	2 (50.0%)	2 (50.0%)	
	2+	4 (9.3%)	3 (75.0%)	1 (25.0%)	
	3+	3 (7.0%)	2 (66.7%)	1 (33.3%)	
**MUC1, *n* (%)**	Negative	10 (23.3%)	5 (50.0%)	5 (50.0%)	0.481
	Positive	33 (76.7%)	12 (36.4%)	21 (63.6%)	
**MUC2, *n* (%)**	Negative	28 (65.1%)	14 (50.0%)	14 (50.0%)	0.101
	Positive	15 (34.9%)	3 (20.0%)	12 (80.0%)	
**COX2, *n* (%)**	Negative	11 (25.6%)	3 (27.3%)	8 (72.7%)	0.480
	Positive	32 (74.4%)	14 (43.8%)	18 (56.2%)	
**panCK, *n* (%)**	Negative	21 (48.8%)	12 (57.1%)	9 (42.9%)	**0.031**
	Positive	22 (51.2%)	5 (22.7%)	17 (77.3%)	
**Surgery performed, *n* (%)**	No	6 (14.0%)	0 (0.0%)	6 (100.0%)	0.066
	Yes	37 (86.0%)	17 (45.9%)	20 (54.1%)	
**Type of surgery, *n* (%)**	Total gastrectomy	29 (78.4%)	14 (48.3%)	15 (51.7%)	1.000
	Proximal subtotal	3 (8.1%)	1 (33.3%)	2 (66.7%)	
	Distal subtotal	4 (10.8%)	2 (50.0%)	2 (50.0%)	
	Palliative	1 (2.7%)	0 (0.0%)	1 (100.0%)	
**Extent of dissection, *n* (%)**	D0	1 (2.7%)	0 (0.0%)	1 (100.0%)	1.000
	D1	2 (5.4%)	1 (50.0%)	1 (50.0%)	
	D2	34 (91.9%)	16 (47.1%)	18 (52.9%)	
**Resection status, *n* (%)**	R0	26 (70.3%)	15 (57.7%)	11 (42.3%)	**0.044**
	R1	10 (27.0%)	2 (20.0%)	8 (80.0%)	
	R2	1 (2.7%)	0 (0.0%)	1 (100.0%)	
**Surgical margin, *n* (%)**	Negative	26 (70.3%)	15 (57.7%)	11 (42.3%)	**0.036**
	Positive	11 (29.7%)	2 (18.2%)	9 (81.8%)	
**Total number of LNs removed** Mean ± SD/Median (Min–Max)		21.36 ± 12.38/17.5 (4–52)	23.94 ± 12.13/22 (9–52)	19.05 ± 12.47/15 (4–48)	0.127
**Metastatic LNs** Mean ± SD/Median (Min–Max)		4.00 ± 8.01/0.5 (0–38)	1.35 ± 2.87/0 (0–9)	6.37 ± 10.24/2.0 (0–38)	**0.019**

Abbreviations: *n*, Number of patients; IHC, Immunohistochemistry; HER2, Human epidermal growth factor receptor 2; MUC, Mucin; COX2, Cyclooxygenase-2; panCK, Pan-cytokeratin; LN, Lymph node; R, Resection (surgical margin status).

**Table 3:** Neoadjuvant therapy characteristics and relationship with outcome.

		Total	Alive	Deceased	*p*
**Neoadjuvant ECOG, *n* (%)**	0	28 (65.1%)	13 (46.4%)	15 (53.6%)	0.367
	1	13 (30.2%)	4 (30.8%)	9 (69.2%)	
	2	2 (4.7%)	0 (0.0%)	2 (100.0%)	
**Neoadjuvant chemotherapy regimen, *n* (%)**	FLOT	20 (46.5%)	12 (60.0%)	8 (40.0%)	**0.014**
	Other	23 (53.5%)	5 (21.7%)	18 (78.3%)	
**Neoadjuvant toxicity, *n* (%)**	No	5 (11.6%)	4 (80.0%)	1 (20.0%)	0.071
	Yes	38 (88.4%)	13 (34.2%)	25 (65.8%)	
**Neoadjuvant toxicity (Grade 1–2), *n* (%)**	0	4 (9.3%)	4 (100.0%)	0 (0.0%)	**0.019**
	1	39 (90.7%)	13 (33.3%)	26 (66.7%)	
**Neoadjuvant toxicity (Grade 3–4), *n* (%)**	0	35 (81.4%)	14 (40.0%)	21 (60.0%)	1.000
	1	8 (18.6%)	3 (37.5%)	5 (62.5%)	
**Neutropenia (Grade 0–4), *n* (%)**	0	22 (51.2%)	10 (45.5%)	12 (54.5%)	0.908
	1	7 (16.3%)	2 (28.6%)	5 (71.4%)	
	2	7 (16.3%)	2 (28.6%)	5 (71.4%)	
	3	4 (9.3%)	2 (50.0%)	2 (50.0%)	
	4	3 (7.0%)	1 (33.3%)	2 (66.7%)	
**Anemia (Grade 0–2), *n* (%)**	0	9 (20.9%)	7 (77.8%)	2 (22.2%)	**0.016**
	1	23 (53.5%)	8 (34.8%)	15 (65.2%)	
	2	11 (25.6%)	2 (18.2%)	9 (81.8%)	
**Neuropathy (Grade 0–2), *n* (%)**	0	41 (95.3%)	16 (39.0%)	25 (61.0%)	0.640
	1	1 (2.3%)	0 (0.0%)	1 (100.0%)	
	2	1 (2.3%)	1 (100.0%)	0 (0.0%)	
**Diarrhea (Grade 0/1), *n* (%)**	0	41 (95.3%)	16 (39.0%)	25 (61.0%)	1.000
	1	2 (4.7%)	1 (50.0%)	1 (50.0%)	
**Neoadjuvant treatment discontinuation, *n* (%)**	No	41 (95.3%)	17 (41.5%)	24 (58.5%)	0.511
	Yes	2 (4.7%)	0 (0.0%)	2 (100.0%)	
**Neoadjuvant clinical response, *n* (%)**	PD	5 (11.6%)	0 (0.0%)	5 (100.0%)	**0.010**
	SD	5 (11.6%)	0 (0.0%)	5 (100.0%)	
	PR	32 (74.4%)	16 (50.0%)	16 (50.0%)	
	CR	1 (2.3%)	1 (100.0%)	0 (0.0%)	
**Neoadjuvant pathologic response, *n* (%)**	CR	5 (11.6%)	2 (40.0%)	3 (60.0%)	**0.042**
	PR	27 (62.8%)	14 (51.9%)	13 (48.1%)	
	Poor/No	11 (25.6%)	1 (9.1%)	10 (90.9%)	

Abbreviations: ECOG, Eastern Cooperative Oncology Group performance score; FLOT, 5-fluorouracil, leucovorin, oxaliplatin and docetaxel regimen; PD, Progressive disease; SD, Stable disease; PR, Partial response; CR, Complete response.

**Table 4:** Comparison of laboratory variables in the total cohort, alive, and deceased groups.

	Total (Median [Min–Max])	Alive (Median [Min–Max])	Deceased (Median [Min–Max])	*p*
**Pre-treatment Hb (g/dL)**	11.9 (7.8–16.3)	11.6 (7.8–15.9)	12.0 (8.4–16.3)	0.973
**Pre-treatment RDW (%)**	14.80 (10.5–29.6)	14.80 (10.5–29.6)	14.75 (11.3–25.0)	0.737
**Neutrophils (10^3^/mm^3^)**	5292 (1.580–10.800)	5320 (2.765–10.400)	5070 (1580–10,800)	0.576
**Monocytes (10^3^/mm^3^)**	610 (134–1490)	652 (0.179–1.024)	546.5 (134–1490)	0.590
**Lymphocytes (10^3^/mm^3^)**	2040 (0.168–22.410)	2230 (1200–22,410)	1765 (168–3370)	0.238
**Platelets (10^3^/mm^3^)**	283 (165–559)	285.6 (175.2–559)	281 (165–548)	0.502
**Albumin (g/dL)**	4.1 (2.6–4.6)	4.0 (2.7–4.6)	4.1 (2.6–4.6)	0.636
**LDH (U/L)**	185 (115–1559)	185 (136–285)	183 (115–1559)	0.813
**CEA (ng/mL)**	2.20 (0.60–155.00)	1.60 (0.70–9.70)	2.75 (0.60–155.00)	**0.014**
**CA 19-9 (U/mL)**	35.0 (2–1596)	17.0 (2–709)	154.5 (2–1596)	**0.044**
**Ki-67 (%)**	70 (10–80)	70 (10–80)	65 (20–80)	0.574
**Post-neoadjuvant CEA (ng/mL)**	3.00 (0.6–30.6)	2.60 (0.6–7.1)	3.50 (0.9–30.6)	0.320
**Post-neoadjuvant CA 19-9 (U/mL)**	14.00 (1–1555)	8.00 (1–92.5)	12.00 (2–1555)	**0.040**
**NLR**	3.00 (0.6–30.6)	2.60 (0.6–7.1)	3.50 (0.9–30.6)	0.728
**PLR**	137.27 (9.1–1684.5)	126.82 (9.1–295.0)	164.10 (59.1–1684.5)	0.585
**MLR**	0.291 (0.03–3.80)	0.303 (0.03–0.47)	0.290 (0.05–3.80)	0.941
**SII**	671.24 (30.53–8944.82)	829.88 (30.53–2049.65)	651.99 (147.73–8944.82)	0.823
**SIRI**	1.51 (0.10–20.17)	1.56 (0.10–3.92)	1.44 (0.18–20.17)	0.691
**PNI**	18.74 (0.85–239.71)	27.37 (5.74–239.71)	17.72 (0.85–82.88)	0.502
**PIV**	388.55 (19.90–7976.02)	474.20 (19.90–1447.27)	356.33 (65.44–7976.02)	0.188
**HRR (Hb/RDW)**	0.840 (0.36–1.38)	0.840 (0.36–1.38)	0.840 (0.38–1.31)	0.970

Abbreviations: Min, Minimum; Max, Maximum; Hb, Hemoglobin; RDW, Red cell distribution width; LDH, Lactate dehydrogenase; CEA, Carcinoembryonic antigen; CA 19-9, Carbohydrate antigen 19-9; NLR, Neutrophil-to-lymphocyte ratio; PLR, Platelet-to-lymphocyte ratio; MLR, Monocyte-to-lymphocyte ratio; SII, Systemic immune-inflammation index; SIRI, Systemic inflammation response index; PNI, Prognostic nutritional index; PIV, Pan-immune-inflammation value; HRR, Hemoglobin/RDW ratio.

## Data Availability

The data that support the findings of this study are available on request from the corresponding author, S.O.O. The data are not publicly available due to ethical reasons.
